# Successful Reach and Adoption of a workplace health promotion RCT targeting a group of high-risk workers

**DOI:** 10.1186/1471-2288-10-56

**Published:** 2010-06-14

**Authors:** Marie B Jørgensen, Charlotte DN Rasmussen, Dorte Ekner, Karen Søgaard

**Affiliations:** 1 The National Research Centre for the Working Environment, Copenhagen, Denmark; 2 Institute of Sports and Exercise Science, University of Copenhagen, Denmark; 3 Institute of Sports Science and Clinical Biomechanics, University of Southern Denmark, Denmark

## Abstract

**Background:**

Cleaners are rarely introduced to workplace health promotion programs. The study's objective was to evaluate the reach and adoption of a workplace randomized controlled trial (RCT) among cleaners in Denmark.

**Methods:**

Cleaning businesses with at least 30 employees, that could offer a weekly 1-hour intervention during working hours, were invited to participate. Employees working at least 20 hours/week were invited to answer a screening questionnaire and consent to participate. Analyses determined the differences in health variables between responders and non-responders, consenters and non-consenters, participants and non-participants and between participants of the RCT's three groups: physical coordination training, cognitive-behavioural theory-based training and reference group.

**Results:**

From 16 eligible workplaces, a representative sample of 50% adopted the trial. Of 758 eligible employees, 78% responded to the screening questionnaire and 49% consented to participate. Consenters and participants differed from non-consenters and non-participants by having higher BMI, more chronic diseases and poorer musculoskeletal health.

**Conclusions:**

This study indicates that workplace health promotion programs directed at health risk factors among cleaners enable significant adoption and reach to a high-risk subgroup of the Danish workforce.

**Trial registration:**

Trial registration ISRCTN96241850

## Background

Cleaners represent a group of mostly non-educated, multi-ethnic, low-wage workers with an elevated risk of musculoskeletal symptoms, cardiovascular diseases[1] and early retirement from the labour market[2]. It has been shown that workplace health promotion programs are offered less to people with poor education than they are to more highly educated people[3]. Furthermore, multi-ethnic low-wage groups are less likely to participate in public health programs[4-6]. Health promotion programs aimed at cleaners may therefore serve as a legitimate attempt to reduce class-based health disparities by directly targeting a health-challenged group.

In 1987, Conrad[7] stated that healthy employees who were the least in need of exposure to health promotion were more likely to participate in worksite health promotion programs. For several years, researchers have sought a better evaluation of *who does *and *who doesn't *participate in intervention studies in order to identify one determinant of such studies' external validity[8-13]. Despite this, reports of external validity are lacking in many trials[6,10,12]^. ^The RE-AIM framework has been introduced as a means to systematically describe determinants of external validity[12]. RE-AIM includes five dimensions of quality:1) Reach 2) Efficacy 3) Adoption, 4) Implementation and 5) Maintenance.

Many employed cleaners appear to balance on the borderline of being either patients or workers, probably using their sick leave days as a coping strategy to continue working. Such high-risk groups are usually not reached before they present for clinical treatment. A recent study showed that participants in a health promotion program drawn from workers with an elevated risk of cardiovascular disease had a less favourable health profile than non-participants[14]. Using the workplace as an arena for health promotion therefore introduces the possibility of conducting preventive programs for high-risk workers - hopefully before they become patients. Therefore, the reach of this kind of intervention to workers in cleaning workplaces is interesting, especially if and how those participating differ from the non-participants.

A randomized controlled trial (RCT) was set up in the identified cleaners' workplaces to test the effect of cognitive behavioural therapy-based training (CBT) and physical coordination training (PCT) on cleaners' work ability, musculoskeletal symptoms and sick leave. Both CBT and PCT have in other settings proved effective in reducing musculoskeletal symptoms[15-23]. The focus in the current study was on reach and adoption of the RCT. These are two of the RE-AIM dimensions: 'reach' defined as the representativeness of the participants, and 'adoption' defined as the percentage and representativeness of the workplaces that consented to participate in the study. Furthermore, to add detail to the analysis of reach, an examination of the disparity between the intervention groups and the control group was conducted. Information on the three remaining RE-AIM dimensions of efficiency, implementation and maintenance will be collected at a later date and are therefore beyond the scope of this article.

The aim of this study was to evaluate the reach and adoption of a workplace health promotion intervention among cleaners in Denmark. Data on the reach of the study was obtained through questionnaire responses from 78% of the eligible employees of cleaning work places and data on adoption relied on a thorough description of the workplace recruitment procedure.

## Methods

The study was a cluster randomized controlled intervention conducted at cleaning workplaces in Denmark with the overall goal of determining if the intervention could improve work ability, musculoskeletal symptoms and sick leave among cleaners. The study was of one year's duration and conducted in two rounds. The first round ran at three workplaces from September 2007 to September 2008, and the second at six workplaces from October 2008 to October 2009. Ethics approval was received from the local ethics committee (H-C-2007-0033) and the RCT was registered with a unique trial registration number (ISRCTN96241850). Details regarding the overall concept and design of the study are given in Holtermann et al. 2010 [24].

### Target population

Participants in this study come from the Danish capital, Copenhagen, and from within a 100 km radius of Copenhagen. They were recruited from managers' lists of employees, which included their civil registration number with linked information about age and sex. All potential participants were invited to answer a screening questionnaire on age, sex, height, weight, working hours, job seniority, leisure time physical activity (LTPA), diseases and musculoskeletal disorders as well as a request for their consent to participate in the RCT. These data allow for analysis of differences between consenters and non-consenters as well as participants and non-participants in the RCT.

### Workplace recruitment

Hospitals, cleaning companies, and large businesses with in-house cleaning services situated in the target area, were identified through internet search, union and company networks, or from common knowledge in the research department. In order to be able to randomize participants into clusters, an inclusion criterion for a workplace was that at least 30 cleaning employees were required to be engaged in the same geographical area. Furthermore, workplaces needed to be able to offer the intervention either as part of the employees' working day or give the employees an opportunity to be compensated with overtime when it was spent participating in the interventions.

### Recruitment procedure

The lists of employees obtained from managers were thoroughly examined to check for their eligibility. Participants were required to be employed for at least 20 hours/week at the workplace. Their main work task had to be cleaning, but their job could also involve other service tasks such as washing, kitchen work or attending to patients. Furthermore, participants were required to work primarily during day hours.

All eligible employees were invited to an information meeting during their working hours and asked to fill out a screening questionnaire and to give consent (consenters) or not (non-consenters) to enrol in the study. For employees who did not attend the information meeting, managers subsequently handed them written information on the project and screening questionnaires with a stamped addressed envelope. Consenters were then invited for physical testing and questionnaire sessions during working hours. For safety reasons, exclusion criteria (pregnancy, diagnosed angina pectoris, life-threatening diseases) were introduced since maximal strength testing was part of the evaluation.

During this initial phase, the employees could withdraw from the study if they had changed their mind regarding consent. Non-consenters had no further contact with the study personnel. Employees accepted for participation in the study at the end of testing were enrolled and randomly allocated to one of three groups: PCT, CBT or Reference group [24].

### Randomization procedure

For the cluster randomization procedure, each workplace was considered a stratum. Clusters depended on work teams where possible or were made up from groups either in which employees had lunch, groups where they worked in close proximity to each other, or groups who reported to the same manager. Clusters were matched on sex, age and job seniority. The randomization was made by lot by blinded staff.

### Intervention study design

The interventions were conducted during working hours by research staff and were comprised of three phases: Phase 1 was intensive in terms of time spent on the intervention (1 hour/week) and lasted 3 months; Phase 2 was a transition phase, lasting another 3 months, where the intervention time was gradually reduced from that in Phase 1 to reach the intervention level of Phase 3; and Phase 3 was the least intensive (1 hour/month) lasting 6 months.

#### Physical Coordination training

PCT was conducted in groups of 6-8 participants. The aim was to change the relation between work demands and capacity, thereby decreasing the relative physical load during work. Exercises to strengthen stability and coordination of trunk and shoulder muscles were conducted at an intensity level corresponding to 50-80% of maximal muscle activity [25]. In Phase 1, the training involved 3 sessions per week, each lasting 20 minutes; in Phase 2, 1-2 sessions per week, each of 20 minutes duration; and in Phase 3, one session every month lasting one hour.

#### Cognitive behavioural theory-based training

CBT was conducted in groups of 6-8 participants led by instructors with a physiotherapy or physiology background, who had been specifically educated in CBT. The participants were educated in discriminating injury from muscle pain when choosing appropriate individual coping strategies. When relevant, also possible changes in organisation of work tasks as coping strategy were considered. During Phase 1, sessions every second week lasted 2 hours, following a modified version of an intervention program developed by Linton[26]. During Phase 2, monthly sessions lasted 2 hours and during Phase 3, monthly sessions lasted 1 hour.

#### Reference group

Participants in the reference group were offered a one-hour health check, performed by a physiotherapist following randomization. Participants received immediate feedback on their results.

### Outcome measures

The RCT was evaluated by questionnaires and physical testing at baseline, after three months and after one year by research staff not involved in the interventions. Further description of the outcome measures for the RCT has been documented elsewhere [24]. Information for analysing differences between questionnaire responders and non-responders was obtained from the managers' lists of employees, which contained employees' civil registration number with date of birth and sex.

To evaluate the representativeness of each group recruited for the study, information on demographics and health were obtained from the questionnaire. The demographic data consisted of age, sex, height, body weight, participants' working hours, job seniority and level of LTPA. Participants' working hours were assessed by the question : "How many hours a week do you normally work in your principal occupation, including paid overtime, work undertaken at home, and other kinds of extra work?". The question on job seniority posed was: "How long have you been employed in your current job or in a job with similar tasks?" which was measured in years. To obtain information on the participants' LTPA, Saltin and Grimby's validated questionnaire was used[27]. The following question was posed: "Looking back over the past year, what would you say fits best with your spare time activity: (i) Almost totally physically inactive or lightly physically active for less than 2 hours per week (e.g. reading, television, cinema), (ii) Lightly physically active for 2-4 hours per week (e.g. walking, bicycling, easy gardening, easy gymnastics), (iii) Lightly physically active for more than 4 hours per week or more strenuously physically active for 2-4 hours per week (e.g. fast walking, bicycling i.e. overtaking others, heavy gardening, strenuous gymnastics causing sweating and losing your breath) (iv) More strenuous physical activity for more than 4 hours per week or regular heavy training and possibly competition several times per week".

The health data consisted of information on four different physician-diagnosed injury or disease-groups: injury from accidents, musculoskeletal diseases, cardiovascular diseases and respiratory diseases. Information on four different injury or disease groups: injury from accidents, musculoskeletal diseases, cardiovascular diseases and respiratory diseases were collected via the following question [28]: "Do you suffer from the following injuries or diseases? Also indicate whether a physician has diagnosed or treated these diseases (for each injury or disease there can be 1 or 2 alternatives answered. 1) Problems due to previous injury/injuries (example - damage to head/neck, shoulders or back) 2) Musculoskeletal diseases (example - rheumatoid arthritis in the neck, shoulders or low back) 3) Cardiovascular diseases (examples - hypertension, chest pain during exercise, cardiac insufficiency) 4) Respiratory diseases (examples - repeated infections of the respiratory tract, bronchial asthma, pulmonary enlargement) (No/yes, own opinion/yes, physician's diagnosis). For the statistical analysis, the number of injuries and diseases was calculated from only the answers "yes and physician's diagnosis" (0-4 diseases).

Musculoskeletal symptoms during the previous 12 months were reported according to the Nordic Questionnaire on Trouble [29]. The following questions were posed: "How many days have you had trouble in [body part] during the last 12 months?" (0 days, 1-7 days; 8-30 days; >30 days; every day) for the duration of the symptoms. The questions posed in relation to the body part were firstly the neck, then shoulders, then upper back and then low back. For statistical analysis, the scale on musculoskeletal symptoms was dichotomized so that "0 days", "1-7 days" and "8-30 days" were categorized as healthy and ">30 days" and "every day" were categorized as workers with chronic pain.

### Statistical analysis

When comparing respondents with non-respondents to the screening questionnaires, consenters with non-consenters, and participants with non-participants, a Student's t-test was conducted for age, BMI, job seniority and working hours. Pearson's chi^2 ^was used to test for differences in sex distribution, and the dichotomized parameter for musculoskeletal symptoms in neck, shoulders, upper back and lower back. A Mann-Whitney test was conducted to test for differences in LTPA and number of diseases.

When comparing the three intervention groups, one-way analysis of variance (ANOVA) was conducted to test for age, BMI, job seniority and working hours. Pearson's chi^2 ^was used to test for differences in sex distribution, and the dichotomized parameter for musculoskeletal symptoms in neck, shoulders, upper back and lower back. The Kruskal-Wallis test was used to test differences in LTPA and number of diseases. SPSS (version 17.0) statistical software was used for the statistical analysis.

## Results

### Workplace recruitment

**Figure 1:**
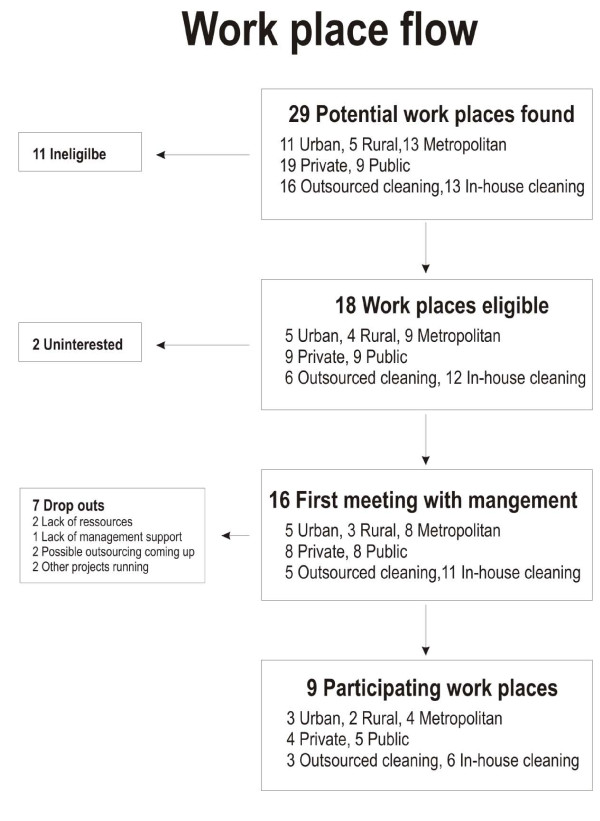
**Workplace Recruitment**. Flow chart on workplace recruitment and adoption with identification of workplace characteristics.

Twenty-nine potential workplaces were contacted during March-June 2007 and March-June 2008. Of these, approximately two-thirds were private and one-third were public companies. Eleven workplaces did not meet the inclusion criteria on number of employees. Of the 18 remaining workplaces, 16 were interested in further information at a meeting with 50%/50% private/public companies, 31.3%/18.7%/50% from urban/rural/metropolitan areas, and 31.3%/68.7% outsourced/in-house cleaning workplaces, respectively. The two workplaces not interested in receiving further information were private/public, outsourced/in-house cleaning and rural/metropolitan, respectively. The 16 workplaces were thoroughly informed about the study during separate meetings with their management teams. Then, 7 workplaces withdrew for different reasons: the possibility of being outsourced (2 private/public, rural/metropolitan, in-house cleaning), lack of management support (1 private, metropolitan, outsourced), lack of resources (2 public, urban/metropolitan, in-house cleaning) or other projects running at the same time (2 private, metropolitan/urban, in-house cleaning/outsourced). In all, nine workplaces enrolled in the study. These nine workplaces were well matched with the 18 eligible workplaces with respect to the distribution of public/private companies, location of workplace and organisational design. The workplace recruitment process is shown in Figure 1.

### Participant recruitment

**Figure 2:**
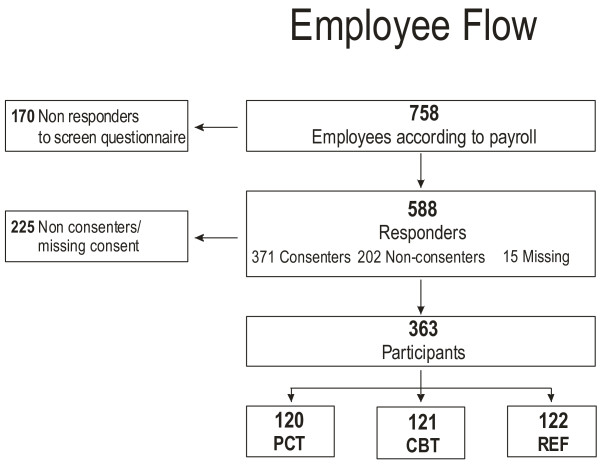
**Employee Flow**. Flow chart on employee recruitment and reach. PCT = Physical coordination training group, CBT = Cognitive behavioural training group, REF = Reference group.

In a number of cases, the initial employee list made available was not up-to-date concerning employee termination or new employment contracts. The proportion of employees deemed eligible for inclusion in the study was 83%, with five hundred and eighty-eight people responding to the questionnaire (Figure 2). The proportion of participants enrolled after testing of those deemed eligible was 47.9%.

### Responders versus non-responders to screening questionnaires

There were no differences in age and sex distribution among responders (n = 588) and non-responders (n = 148) to the questionnaire. Women comprised 81% and 78% among the responders and non-responders respectively. Mean age (standard deviation (SD)) was 44.8(10.0) and 42.8(12.0) years for the responders (n = 582) and non-responders (n = 169), respectively.

### Consenters versus non-consenters to the screening questionnaire

There were no differences between consenters (n = 371) and non-consenters (n = 202) in the questionnaire on age, sex, job seniority, weekly working hours and LTPA (Table 1). Fifteen responders did not answer the question in the screening questionnaire regarding consent, and were therefore excluded.

**Table 1:** Group characteristics.

		Consenters	Non-consenters	**Level of sign**.	Participants	Non-participants	**Level of sign**.
		(n = 371)	(n = 202)	p	(n = 363)	(n = 395)	P
Age (Years)	N	371	198		363	390	
	**mean**	**44.7**	**44.9**	**ns**	**44.8**	**43.9**	**ns**
	sd	9.4	11.1		9.3	11.5	

Sex (Females)	N	371	201		363	372	
	**%**	**81**	**81**	**ns**	**81**	**80**	**ns**

BMI (kg/m^2^)	N	323	163		312	185	
	**mean**	**26.8**	**25.6**	**<.05**	**26.9**	**25.5**	**<.01**
	sd	4.9	5.1		4.8	5.0	

Job seniority (Years)	N	300	150		290	171	
	**mean**	**9.1**	**10.1**	**ns**	**9.2**	**10.0**	**ns**
	sd	8.6	9.6		8.5	9.7	

Working hours (Hours/week)	N	338	191		327	213	
	**mean**	**34.9**	**34.0**	**ns**	**34.7**	**34.2**	**ns**
	sd	6.3	7.3		6.7	6.9	

PHA (1-4)	N	305	149		294	167	
	**mean**	**2.3**	**2.3**	**ns**	**2.3**	**2.3**	**ns**
	sd	0.9	0.9		0.9	0.9	

Diseases (1-4)	N	112	62		111	63	
	**mean**	**0.7**	**0.3**	**<.01**	**0.7**	**0.3**	**<.01**
	sd	1.0	0.6		1.0	0.6	

Characteristics of consenters versus non-consenters and participants versus non-participants.

n = number of responders, sd = standard deviation, ns = non-significant to the level of p = 0.05.

**Figure 3:**
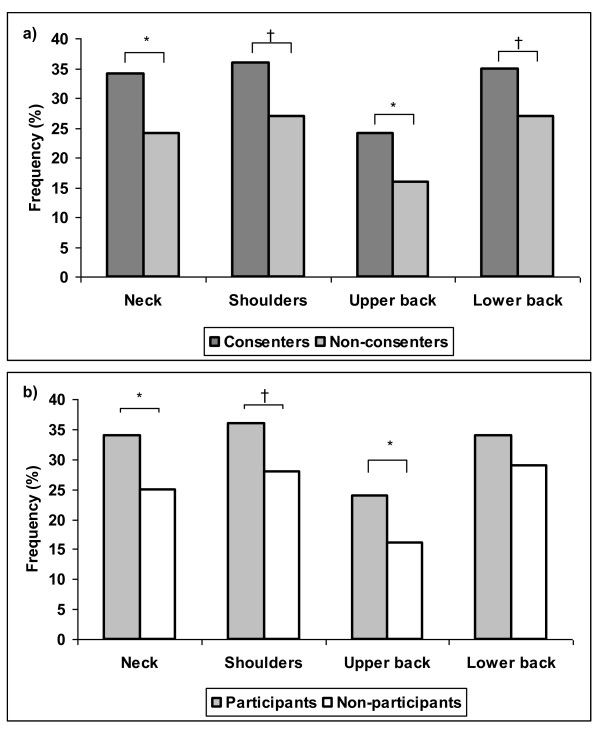
**a+b - Musculoskeletal symptoms**. Proportion of employees with musculoskeletal symptoms above 30 days during the past year among a) consenters versus non-consenters and b) participants versus non-participants. † = p < 0.1, * = p < 0.05.

The consenters though, had a significantly higher BMI (26.8) compared with the non-consenters (25.6). A larger proportion of consenters had chronic pain in both the neck and upper back, and a history of more chronic pain in their shoulders and lower back during the previous year than the non-consenters (Figure 3a). Finally, this group suffered from significantly more diseases than the non-consenters as summarized in Table 1.

### Participants versus non-participants in the RCT

When comparing the same parameters in participants versus non-participants following randomization, the same results were found with two exceptions (Table 1). Firstly, there was no trend showing a difference in the percentage of workers with chronic low back pain (Figure 3b) and the difference in BMI was statistically stronger.

### Randomization and comparison of the intervention groups

The three randomized groups consisted of 120, 121 and 122 participants in PCT, CBT and REF, respectively. The mean(SD) for age was 44.4(8.9)/45.7(9.0)/44.1(9.9), for BMI was 27.0(5.0)/26.8(5.1)/26.8(4.6), for working hours was 35.1(6.3)/33.7(8.5)/35.1(4.9), for job seniority was 8.8(8.6)/8.9(7.7)/9.7(9.2), for number of illnesses was 0.5(0.7)/0.8(1.0)/0.9(1.1), and the median LTPA was 2.0/2.0/2.0 for the PCT/CBT/REF, respectively. The percentage of women was 79.2/81.8/82.0 for the PCT/CBT/REF, respectively. Finally the proportion of workers with chronic pain in the neck was 34.3/32.3/34.9, in the shoulders 32.7/41.4/34.5, in the upper back 27.6/22.8/22.0 and in the low back 36.8/33.0/33.1 for the PCT/CBT/REF, respectively. There were no statistical differences between the randomized groups on any variables tested.

## Discussion

This study indicates that a workplace health promotion program directed at health risk factors in the target group of cleaners enables significant reach to a high-risk subgroup of the workforce. The adoption rate of this RCT was 50% of eligible workplaces. This is higher than the 20% adoption rate reported by Barbeau and co-workers[3] in cancer prevention research trials at the workplace. The adoption rate in the current study is more comparable with that found by Bull and co-workers[6] on general health promotion research programs at the worksite (56.5%). Other studies have used large databases in search of eligible workplaces, which deliver a more objective picture of potential participating workplaces[30]. The distribution of private and public companies was comparable across workplaces that adopted the health promotion program and those that withdrew from the study. The adoption rate was similar between eligible workplaces and enrolled workplaces, indicating that outsourcing was not a barrier to initiating health promotion activities. However, as two public workplaces declined participation due to pending outsourcing, such a transition period may be a barrier for new projects to be adopted.

Two-thirds of the 29 cleaning businesses were private companies. Most of these failed to meet the inclusion criteria with respect to a minimum number of employees. Grosch et al.[31] found that employees at private companies were offered health promotion programs to a lesser extent than employees at public workplaces. A large number of private companies employ only a minimum number of employees, probably making health promotion activities more expensive to introduce. This is supported by the results from a national survey among a representative sample of US worksites[30] where a clear relationship was found between worksites with more employees and those offering programs.

The current study's eligibility criteria combined with the fact that mostly private companies agreed to participate means that the results of the RCT will be applicable primarily to large companies with a profile similar to the workplaces involved in this study. Matching of the workplaces deemed eligible with those that were finally included in the RCT indicates good representation. Three of the seven workplaces that withdrew from the study, cited internal matters such as lack of resources and management support as the cause. Hence, results of the RCT may not be applicable to such workplaces.

Of the people invited to participate in the RCT, 48% actually did participate. This is comparable with the median participation rates of 33% and 61% found in two recent reviews of several worksite health promotion programs[6,32]. Non-participants did not differ from participants in age, sex, working hours, job seniority or LTPA. This indicates that the participants were a representative sample of cleaning employees with respect to age and work-related factors. A number of studies have found differences in participation rates among men and women [30-33] and across age groups [6,31,33]. Lewis et al.[33] and Robroek et al.[32] found a higher participation rate among women than among men, whereas Grosch et al.[31] found, in their national probability sample of the U.S. civilian population, that health promotion programs which included exercise facilities and screening tests attracted males to a higher degree than women. Robroek et al.[32] found that access to fitness centres encouraged men to the same degree as women. In the current study, both screening tests as well as training programs were offered and a similar proportion of males and females consented to participate.

Several studies found that younger employees participate more than other age-groups[31,33], but another review found that the workers who enrolled in the studies were generally older[6] and this is also the case in the recent study from Groeneveld et al. 2009 [14]. The similarity between consenters/non-consenters and participants/non-participants for both age and sex may be explained by the relatively broad intervention theme, resulting in the programs offered appealing equally to females and males, young and old. Robroek et al.[32] also concluded that multiple component programs generally had higher participation rates, probably causing better reach to the target group. Future data on the effectiveness and implementation phase of the current study may reveal a bias in drop-out rate between age and sex groups.

Non-participants could be differentiated from participants in a number of important health parameters; BMI, diseases and musculoskeletal symptoms in the neck, shoulders and upper back. This indicates that the study appealed to an unhealthier proportion of an already high-risk population of cleaners, which is in clear opposition to the hypothesis of Conrad[7], but is consistent with the findings of a recent study by Groeneveld and co-workers [14]. As previously discussed, the participating component of the target population of a health promotion program is generally healthier and at lower risk than the non-participating component[4,6]. Lewis and co-workers[33] investigated the participation rate in several health promotion activities at one worksite among employees at high and low risk. They found that employees at lower risk tended to participate at a higher rate than employees at high risk. Educational programs however, had a significantly higher participation rate among high-risk groups than low-risk groups[33]. Pelletier[34] suggested that providing individualized risk education for high-risk employees within the context of comprehensive programming is the critical element of worksite interventions. Furthermore, Grosch et al.[31] proposed that specific features of the work environment can encourage involvement in health promotion activities. In the current RCT, the intervention had a general goal of improving health combined with a more specific goal of improving certain work environment factors particularly relevant to cleaners. This may explain why the more unhealthy employees were actually motivated to enrol.

The fact that the participants in the RCT on variables such as age, sex, job seniority and work time were representative of the total cleaning staff at the workplaces eligible for inclusion combined with the fact that they were actually in the high-risk group for musculoskeletal symptoms and diseases, is important when evaluating the potential public health impact of the intervention. Furthermore, the fact that at baseline there were no differences between the three intervention groups, is important for the interpretation of the results in the RCT. The difficulty in reaching the high-risk employees has been reported as a barrier to the success of health promotion programs among 48% of interviewed companies in the 2004 National Health Promotion Survey of US worksites[30].

### Strengths and weaknesses of the study

Strengths of the current study included the high adoption rate among eligible workplaces; the workplaces' characteristics being representative of the target population; the completion of a comprehensive screening questionnaire by a large proportion of those employees eligible for inclusion; and the access given to data which allowed the identification of differences between responders and non-responders in relation to age and sex.

A limitation may have been that the participants in this study were not a totally representative sample of cleaners in Denmark. Therefore, the results of this RCT are not generalizable to all cleaners, but are conclusive in terms of the effect among a high-risk group of cleaners at larger workplaces. Unfortunately, resources were not available for qualitative methods to reveal managers' views on health promotion or for a thorough collection of the eligible but not participating companies' workforce characteristics in relation to age and gender distribution, sick leave prevalence, etc., which presents another limitation of the study.

Efficiency, implication and maintenance, the remaining three RE-AIM dimensions of quality, were not within the scope of this article, but would need to be examined to gain a full picture of this RCT's external validity.

## Conclusions

In this RCT, a high adoption rate of 50% for the workplace health promotion intervention was found among eligible workplaces covering different types of businesses (private/public, urban/rural/metropolitan, outsourced/in-house cleaning). A response rate of 78% was obtained, providing rich information on consenters and non-consenters and participants and non-participants. From the screening questionnaire, consenters and participants in the RCT did not differ from the non-consenters and non-participants, respectively, on age and sex, but differed on important health parameters like BMI, diseases and musculoskeletal symptoms, with consenters and participants showing poorer health. This indicates that it is possible to reach a high-risk group in a workplace when the aim is to improve health and work environment parameters.

## Competing interests

The authors declare that they have no competing interests.

## Authors' contributions

MBJ led the writing of the manuscript, and wrote the first draft of the manuscript. MBJ and KS contributed to the design of the study and all authors contributed to the protocol of the project. DE and CDNR collected and structured the data and contributed to the statistical tests run by MBJ. All authors read and approved the manuscript.

## Pre-publication history

The pre-publication history for this paper can be accessed here:

http://www.biomedcentral.com/1471-2288/10/56/prepub
